# Impact of multivalent charge presentation on peptide–nanoparticle aggregation

**DOI:** 10.3762/bjoc.11.89

**Published:** 2015-05-15

**Authors:** Daniel Schöne, Boris Schade, Christoph Böttcher, Beate Koksch

**Affiliations:** 1 Institute of Chemistry and Biochemistry - Organic Chemistry, Freie Universität Berlin, Takustr. 3, 14195 Berlin, Germany; 2 Electron Microscopy, Freie Universität Berlin, Fabeckstr. 36a, 14195 Berlin, Germany

**Keywords:** coiled-coil peptides, α-helical fibrils, controlled aggregation, gold nanoparticles, multivalency

## Abstract

Strategies to achieve controlled nanoparticle aggregation have gained much interest, due to the versatility of such systems and their applications in materials science and medicine. In this article we demonstrate that coiled-coil peptide-induced aggregation based on electrostatic interactions is highly sensitive to the length of the peptide as well as the number of presented charges. The quaternary structure of the peptide was found to play an important role in aggregation kinetics. Furthermore, we show that the presence of peptide fibers leads to well-defined nanoparticle assembly on the surface of these macrostructures.

## Introduction

In the past few decades metal and semiconductor nanoparticles, including gold nanoparticles, have gained much interest due to their desirable optical, magnetic, and electronic properties [1]. In particular, the distinct colour of gold nanoparticles is a result of the localised surface plasmon resonance (LSPR) band caused by collective electron oscillations. The LSPR induces a certain excitation band at visible wavelengths in the absorption spectrum, the position and width of which is highly dependent upon nanoparticle size. However, nanoparticle aggregation induces a spectral red-shift and broadening of the band in the absorption spectrum which depends on the distance between nanoparticles, the density of the assembly and the size of the particles [2–3]. Thus the controlled assembly of nanoparticles by means of biomolecules is crucial for biological and medical applications such as sensing [4], bioimaging [5], and medical diagnostics [6]. Although nanoparticles are also applied as targeted biomarkers and drug-delivery agents to tumor cells [7], only very little is known about the effects of nanoparticles on whole organisms [8].

Furthermore, there is great interest in using biomolecules as components to build up self-assembled supramolecular organic–inorganic hybrid materials for engineering novel functional materials and molecular devices [9–10]. In spite of the increasing demand for smaller, more complex, but even cheaper materials, the commonly used “top-down” methods are not available. Thus, new strategies like the “bottom-up” approach have been developed to achieve materials in the nanometer range [11–12]. This technique is based on the self-assembly of small building blocks to construct functional materials by means of biomolecules. Recent studies have made use of DNA, lipids, peptides, and proteins to build up organic–inorganic hybrid materials [9]. Due to their versatile and unique functionalities, which can be used for catalytic [13], optics [14], and switching [15] applications, a variety of specific and site selective binding properties are available [16]. In particular, the specificity of Watson–Crick base pairing of DNA nucleotides can be used for the directed and predictable self-assembly of nanoparticles [17]. The DNA mediated assembly of nanoparticles is realized in two different ways. In the first regime, two sets of nanoparticles are functionalized with complementary single-stranded DNA sequences which then anneal to one another [18]; in an alternative setup, adding a complementary linker to nanoparticles functionalised with single-stranded DNA can drive the assembly to form extended networks [19].

Although the relationship between the primary and quaternary structures of peptides and proteins are less clear than for DNA, protein-based recognition systems containing antigen–antibody [20], biotin–streptavidin [21], and peptide–peptide [22] interactions have been explored. In particular, peptide-based assemblies afford numerous advantages such as the modification of nanostructures by mutations of the primary sequence of peptides which may lead to the formation of various hierarchical morphologies [23–25]. The strategies for the assembly of nanoparticles are very similar to those for DNA. Either one part of the recognition system is directly bound to the surface of the nanoparticle by a disulfide bond and the addition of a linker induces assembly, or both linker and acceptor are immobilised on the surface of different nanoparticles and induce assembly [9]. Peptide-based nanoparticle aggregation was demonstrated first by Woolfson and coworkers by means of coiled-coil peptides that were immobilized on the nanoparticle surface [26].

Reversibility of the assembly formation, a key feature of a switchable system, has thus far been explored only for a few nanoparticle systems by means of temperature [19]; most of the assemblies are irreversibly formed [9], or reversibility is only achieved by adding, for example, oxidizing reagents [27]. Continuous switch behaviour between aggregated and non-aggregated nanoparticles is not obtained as the formation of assemblies is most likely achieved by hydrogen bonds or other common receptor–binding interactions [28]. Although it is known that nanoparticles can be organised by binding to membranes by means of electrostatic interactions between the charged head group of the lipid and the oppositely charged nanoparticle [29], only limited effort has been put forth to use peptides or proteins to organize nanoparticles by electrostatic interactions [30–31]. As the overall net charge of a peptide is pH dependent, this can be a powerful tool for the controlled and reversible assembly of nanoparticles.

Recently we published the use of coiled-coil model peptide VW05 for the reversible assembly of mercaptoundecanoic acid functionalized gold nanoparticles using electrostatic interactions [32–33]. We showed that the interaction can be repeatedly switched by adjusting the pH value. We further demonstrated that the ability of the peptide to interact with nanoparticles is directly related to its helical structure and the resulting local charge at the solvent-exposed face of the coiled-coil: a control peptide with the same amino acid composition, which did not follow the regular heptad repeat, was not able to organize nanoparticles in networks. Thus, the electrostatic interaction is not only determined by the overall net charge of the peptide but requires defined spatial ordering and regularity.

Here we report the use of modified peptide variants of the previously studied VW05 for the controlled assembly of gold nanoparticles. As the assembly of charged gold nanoparticles depends on the local charge of the coiled-coil in a multivalent fashion, we wanted to study different aspects of nanoparticle–peptide interactions such as the aggregation tendency of the peptide and the morphology of the obtained peptide–nanoparticle assemblies.

## Results

### Design of the model peptides

The α-helical coiled-coil folding motif combines the chemical diversity of peptides with the molecular recognition properties and structural stability of DNA, and provides a valuable and variable system for the organisation of functionalized nanoparticles [34–36].

The coiled-coil folding motif consists of two to seven α-helices that are wrapped around each other to form a left-handed supercoil. The primary sequence consists of a regular pattern of seven amino acids denoted with a, b, c, d, e, f, and g, which is referred to as the heptad repeat. Positions a and d are commonly occupied by nonpolar amino acids such as leucine or valine to form the hydrophobic core which represents one recognition domain. Amino acids in e and g positions, which flank the hydrophobic core, are often charged and form a second recognition domain due to complementary interhelical electrostatic interactions between the helices. Both recognition domains drive the formation of the coiled-coil, thus they are responsible for the thermodynamic stability of this quaternary structure. Positions b, c, and f, on the other hand, are solvent-exposed and exert only a minor influence on the coiled-coil structure and stability. They are mainly occupied by hydrophilic amino acids and therefore play an important role in interactions with other molecules in the surrounding environment.

In a previous study we showed that the coiled-coil model peptide VW05 induces controlled aggregation of charged gold nanoparticles. The overall primary sequence of VW05 was designed to provide a pH-responsive aggregation of nanoparticles based on electrostatic interactions. Accordingly, a scrambled version of VW05 with the same amino acid composition and net charge did not show any evidence for electrostatic interactions with the nanoparticles and did not trigger nanoparticle aggregation. Thus, it was concluded that the observed nanoparticle–peptide aggregate formation results from the well-defined presentation of four arginine residues in f-positions of the coiled-coil motif [32].

**Figure 1:**
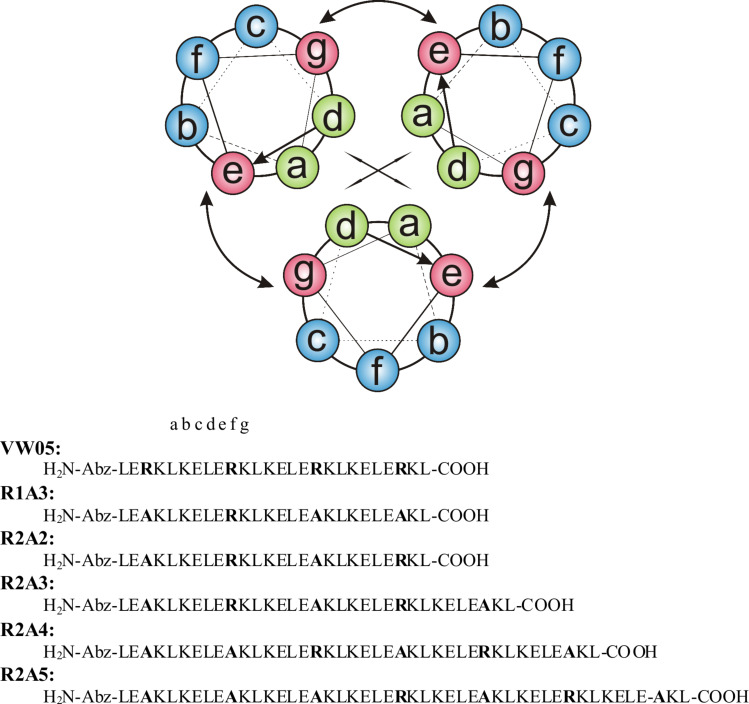
Helical wheel representation and sequences of the peptides used in this study.

In the current study we investigate in detail the effect of the number of presented charges on the aggregation of VW05-based peptides with gold nanoparticles. Therefore, the modified variants R1A3 and R2A2 of the parent peptide, as well as the extended versions R2A3, R2A4, and R2A5, were synthesized and characterized. In the first two cases either three or two arginine residues were substituted with alanine, respectively; alanine is not only neutral, but is also known for its high α-helix propensity. Due to the need of an overall positive net charge of R1A3 and R2A2 to form electrostatic interactions with the nanoparticles, pH 9 is suitable for this study as the calculated overall net charge is positive at this pH value. Taking into account that the assembly of gold nanoparticles may also depend on the length of the peptide and/or on the ratio of presented charges per residue, peptides of greater length are also included in this study by adding one, two, or three heptad repeats containing alanine in their f-positions (R2A3, R2A4 and R2A5); these were based on the sequence of R2A2 (Figure 1).

### Secondary and quaternary structure of the model peptides

**Figure 2:**
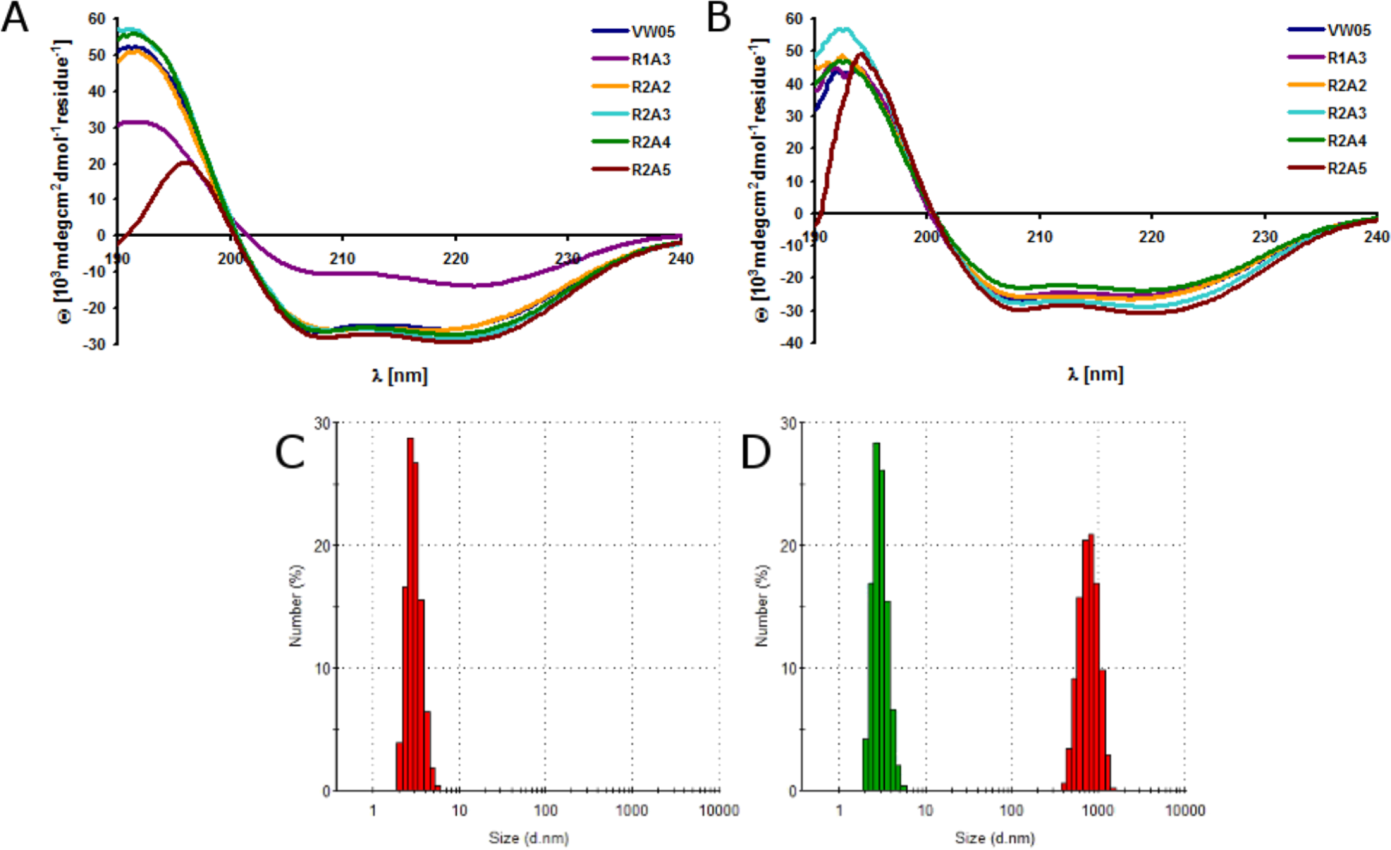
CD spectra of 30 µM peptide VW05, R1A3, R2A2, R2A3, R2A4 and R2A5 at (A) pH 9 and (B) pH 11 in 10 mM Tris/HCl buffer. Dynamic light scattering of (C) VW05 and (D) R2A2 both at 15 µM at (red) pH 9 and (green) pH 11. All measurements were carried out in 10 mM Tris/HCl buffer.

Circular dichroism (CD) spectroscopy and analytical ultracentrifugation of VW05 at pH 9 reveals an α-helical coiled-coil structure that consists of three monomers. Since the modifications of the parent sequence occurred in the solvent-exposed f position only, we expected no differences in the secondary or quaternary structures of peptides R1A3, R2A2, R2A3, R2A4 and R2A5. CD measurements were carried out at pH 9 and pH 11 at a final peptide concentration of 30 µM (Figure 2A/B). The spectra confirmed that all peptides fold into α-helices as indicated by the two characteristic minima at 208 nm and 222 nm and the maximum at 195 nm. Whereas there are no significant differences in the CD spectra at pH 9 of R2A2, R2A3, R2A4, and R2A5, the signal intensity of R1A3 is dramatically decreased. Furthermore, the minimum at 222 nm is increased and this may point to the formation of assemblies containing α-helical fibrils. This is probably a consequence of the formation of peptide fiber bundles which tend to precipitate and thus decrease the concentration. In addition, the fiber bundles may decrease the amount of peptide that is available to generate the CD signal. Increasing the peptide concentration up to 100 µM or incubating samples for periods up to three days do not result in any changes in the CD spectra, indicating that there is no concentration-dependent change in the secondary structure of the peptides. At pH 11 the CD spectra of all peptides are virtually identical and indicate the presence of α-helical coiled-coil structures.

Dynamic light scattering (DLS) was applied to study the oligomerization state of the peptide variants, as analytical ultracentrifugation can not be used for further characterization of peptide fibers due to extreme sample heterogeneity. Since the oligomerization state of VW05 has been studied before [30], the DLS spectrum of VW05 was used as a reference for all other peptides. All measurements were performed at a sample concentration of 15 µM at pH 9 and pH 11 because the net charge of the peptides switches from positive to negative within this pH range. The trimeric coiled-coil VW05 has an average hydrodynamic diameter of approximately 3 nm (Figure 2C). As expected from CD spectroscopy, R1A3 forms α-helical assemblies at pH 9 with an average size of approximately 1 µm but appears to adopt a soluble coiled-coil structure at pH 11 because the particle size decreases to 3 nm. Surprisingly, all other VW05 variants form α-helical assemblies at pH 9 with average sizes of 790 nm (R2A2), 230 nm (R2A3), 180 nm (R2A4), and 160 nm (R2A5). It seems that with increasing sequence length the size of the peptide fibers decreases. This observation is in agreement with a report of Ryadnov and coworkers [37]. Moreover, a second, larger species appears with a size of 1 µm. The occurrence of two fiber species may be the result of competing interactions between arginine residues in f-position and glutamates. This can either stabilize or diminish peptide aggregation. Increasing the pH to 11 leads to a disruption of α-helical peptide fibers that produces coiled-coil monomers. This effect was observed in all cases (Figure 2D).

**Figure 3:**
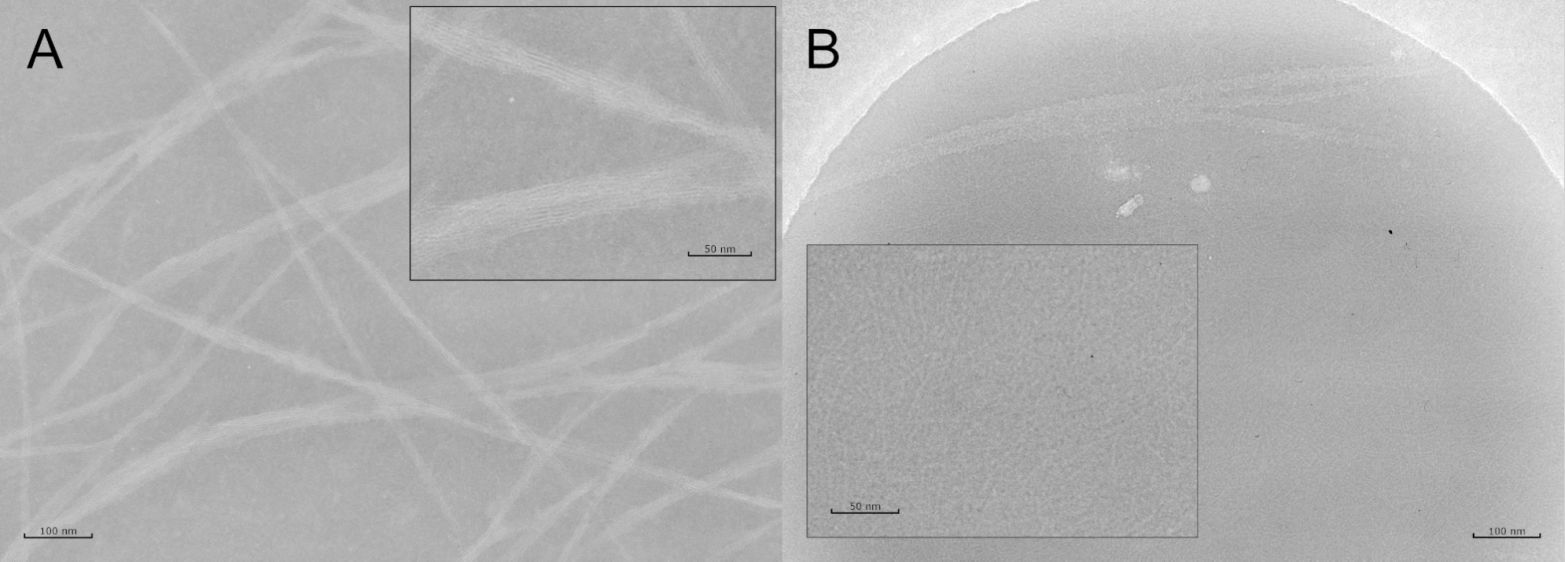
(A) TEM of 100 µM R2A2 in 10 mM Tris/HCl buffer, pH 9. Sample was negative stained with 2% PTA; defocus −0.5 µm. (B) Cryo TEM of 100 µm R2A2 in 10 mM Tris/HCl buffer, pH 9; defocus −1.8 µm.

To resolve the morphology of the quaternary structures, TEM and/or Cryo TEM were applied to all peptides at pH 9 except for VW05; a representative image for R2A2 is shown in Figure 3. Negative staining TEM of 100 µM R2A2 at pH 9 demonstrates the formation of α-helical fibers. Moreover, these fibers appear to form bundles that consist of many long and parallel single fibers; single peptide fibers alone have not been detected. The formed fiber bundles appear to be very rigid in their structure as they appear only as straight and long fibers with a length ranging from several 100 nm up to more than 1 µm. The average diameter of one single fiber is 2.5–3 nm. One single helix has a diameter of 0.5–1 nm, thus one fiber presumably consists of multiple coiled-coil trimers. On the other hand, even single peptide fibers were observed using Cryo TEM; however, they seem to be much shorter in length. However, resolving the microstructure of one fiber bundle using Cryo TEM was not possible. Surprisingly, fiber bundles could not be observed for peptides R2A4 and R2A5 whose sequences were extended by either two or three heptad repeats. It must be concluded that sample drying and the addition of a staining reagent has an effect on the final fiber structure.

### Peptide-induced nanoparticle assembly

The absorption maximum of the LSPR band is a size-dependent property of a gold nanoparticle: the greater the size of the gold nanoparticle, the more red-shifted its absorption maximum. When numerous smaller gold nanoparticles get into close proximity due to aggregation they behave as one larger gold nanoparticle the electronic properties of which can be monitored by the red-shift and broadening of the absorption maximum of the LSPR band. UV–vis absorption spectroscopy was applied to monitor the LSPR band of mercaptoundecanoic acid-functionalized gold nanoparticles (Au/MUA) in the presence of the VW05 variants. Peptides at concentrations ranging from 5 to 30 µM, at pH 9, were added to a nanoparticle solution and UV–vis absorption measurements were carried out. To determine the time-dependence of the assembly process, all measurements were repeated at a three hour time point and a three day time point.

**Figure 4:**
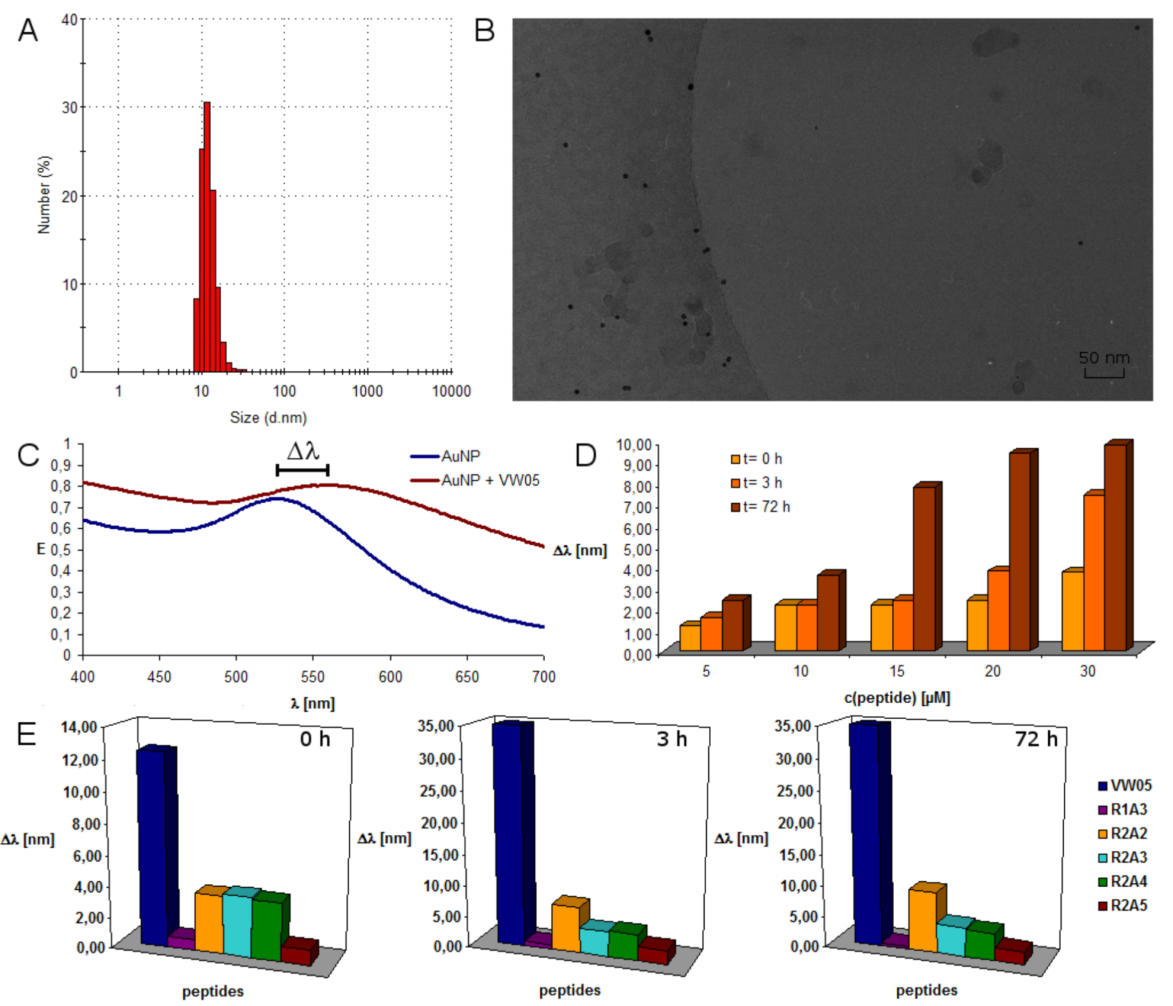
(A) Dynamic light scattering of 0.05 µM Au/MUA nanoparticles at pH 9. (B) Cryo TEM image of 0.05 µM Au/MUA nanoparticles at pH 9. (C) UV–vis spectra of 0.05 µM Au/MUA nanoparticles at pH 9 and in the presence of 30 µM VW05. (D) Time and concentration dependent shift in the absorption maximum of 0.05 µM Au/MUA nanoparticles in the presence of different amounts of R2A2. (E) Time-dependent shift in the absorption maximum at a fixed peptide concentration of 30 µM.

The nanoparticles used in this project were synthesised according to the literature and subsequently refunctionalized in a ligand exchange reaction with mercaptoundecanoic acid [38–39]. The obtained Au/MUA nanoparticles have an average diameter of 5.5 nm, as determined by TEM, and are monodisperse, as confirmed by DLS. An absorption maximum of 525 nm is observed for the Au/MUA nanoparticles in the absence of peptide, even over an incubation time of three days. After addition of the peptide R1A3, only a negligible red-shift of 0.5 nm of the absorption maximum is detected (Figure 4E). Neither an extended incubation time of three days nor an increase in the concentration of the applied peptide to 30 µM leads to a significant increase in the red-shift. This result sharply contrasts with that of the VW05 parent peptide, indicating that the absence of a red-shift and thus a lack of nanoparticle aggregation is very likely a consequence of the reduced number of presented arginine residues in the f-positions of R1A3 compared to VW05. Thus this observation implies that either R1A3 does not interact with Au/MUA nanoparticles or that its interactions are not strong enough to bring the nanoparticles into close proximity to induce a red-shift. Probably the distance between two arginine residues is insufficient for an interaction, which was later on proven by the lack of changes in the secondary structure of peptide R1A3. Accordingly, an increase in the number of arginine residues at f-positions to two in case of R2A2 leads to a significant shift of the LSPR band of 1.20 to 3.75 nm (Figure 4D). As shown in Figure 4D, these red-shifts increase with the peptide concentration as well as with the incubation time; for example, at a concentration of 30 µM of R2A2 the absorption maximum changes by 3.75 nm to 9.80 nm over three days. Although the observed red-shift is minor compared to the parent peptide VW05 at an equal peptide concentration, it can be assumed that incubation with R2A2 leads to an aggregation of Au/MUA nanoparticles. A stepwise increase in the sequence length by adding one heptad repeat without changing the amount of arginine residues (R2A3) leads to a comparable effect (Figure 4E). In contrast, increasing the incubation time to three days does not change the red-shift significantly (Figure 4E); for example, the measured red-shift is 3.75 nm after adding the peptide and 4.70 nm after three days. Adding yet another heptad repeat to the sequence to yield R2A4 affects nanoparticle assembly only marginally, since there is no significant change in the LSPR band compared to R2A3. Even after an incubation time of three days the difference in the red-shift is less than 0.5 nm. Nevertheless, a peptide-induced assembly can be discussed for both peptides. On the other hand, a further increase in the peptide length (R2A5) has a significant effect on the observed LSPR band and thus on nanoparticle aggregation. The determined red-shift of 1 nm is very similar to the observed shift of 0.5 nm for R1A3 measured immediately after adding the peptide. With increasing incubation time the absorption maximum is slightly shifted but even after three days the value is only half of those measured for R2A3 and R2A4.

**Figure 5:**
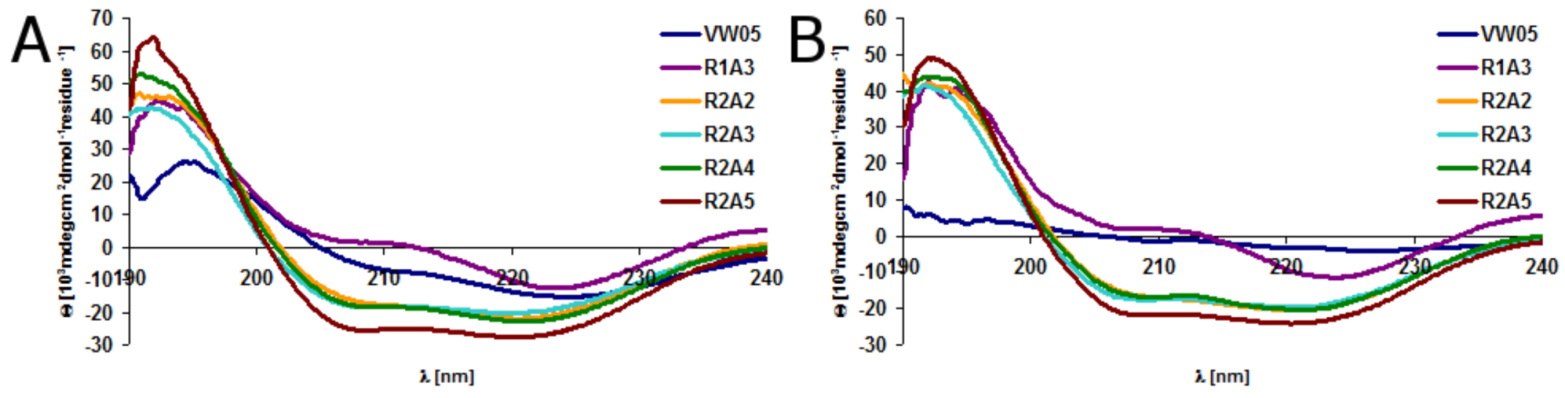
CD spectra of 15 µM peptide in the presence 0.05 µM Au/MUA nanoparticles at pH 9 after (A) 0 hours and (B) three hours.

It is known that gold nanoparticles may affect the secondary structure of a peptide or protein [33]. To investigate the effect of Au/MUA nanoparticles on the secondary structure of the peptides included in this study, CD spectroscopy was applied with a peptide concentration of 15 µM and 0.05 µM Au/MUA nanoparticle concentration. The obtained CD spectra of VW05 show a strong decrease in signal intensity immediately after the addition of Au/MUA nanoparticles (Figure 5A). This can be explained by the almost complete immobilization of the peptide on the nanoparticle surface in multiple layers. Thus, the concentration of dissolved peptide is dramatically decreased and a CD signal can not be detected anymore. A similar effect could be described by Calzolai and coworkers using silver nanoparticles [40]. Furthermore, the minimum at 222 nm increases compared to the minimum at 208 nm which can be attributed to the formation of α-helical fibers. Extending the incubation time to three hours leads to an almost complete loss in the signal intensity of peptide VW05. Peptides R2A2, R2A3, and R2A4 show a somewhat similar effect after incubation with Au/MUA nanoparticles. A decrease in signal intensity as well as an increase in the minimum at 222 nm is observed, although the loss of intensity is not as strong as that observed for VW05. This observation can be attributed to the accumulation of VW05 on the surface of the nanoparticles, whereas the variants form fibrils and do not accumulate in the same way. However, the CD spectra remain stable and no further decrease in the signal intensity is observed during a longer incubation time. CD measurements of R1A3, which induced no red-shift of Au/MUA nanoparticles, reveal no significant structural changes due to nanoparticle addition.

**Figure 6:**
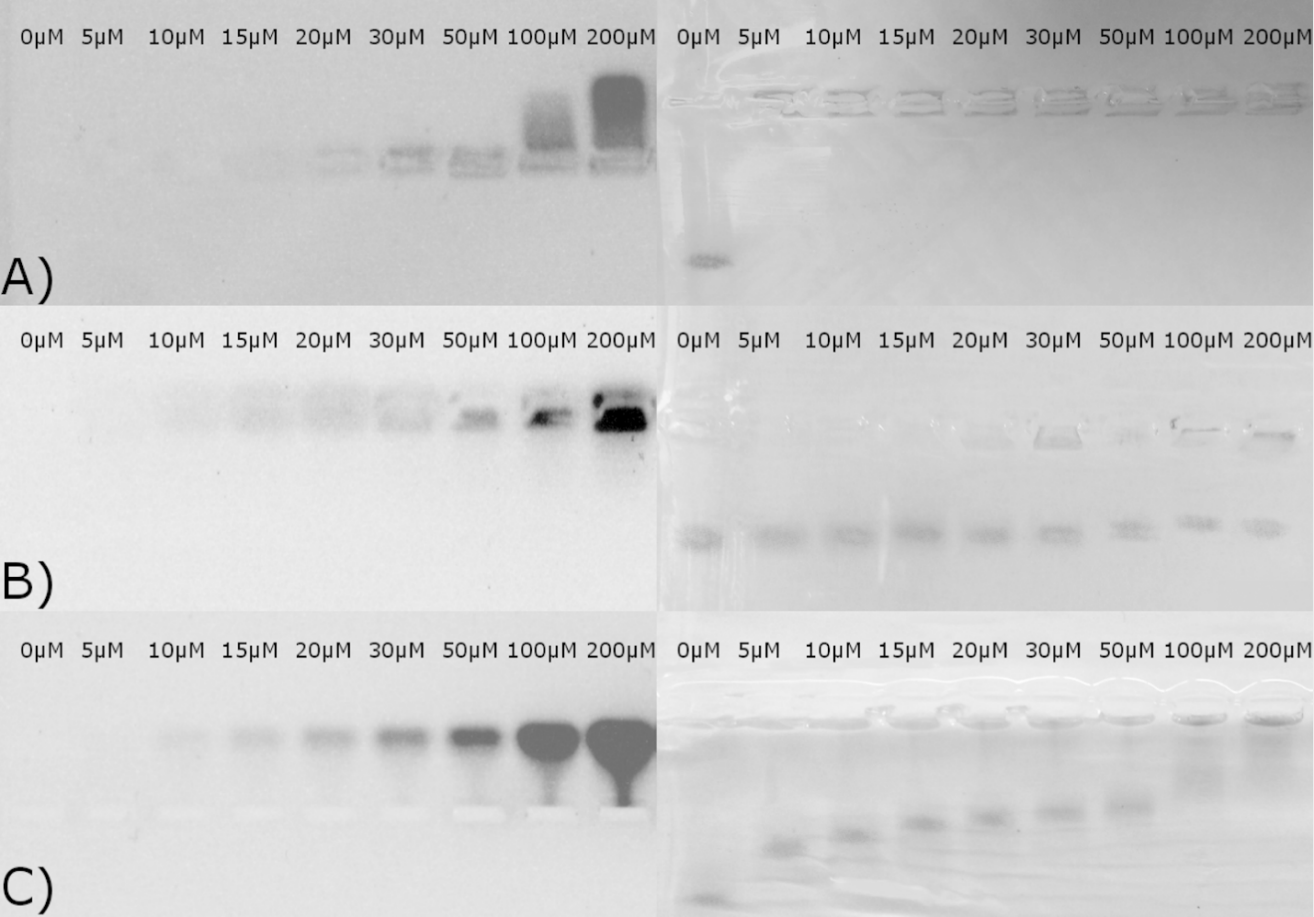
Agarose gel of (A) VW05, (B) R1A3, and (C) R2A2 in the presence of 0.05 µM Au/MUA nanoparticles at pH 9 visualised by UV light (left) and visible light (right).

Agarose gel electrophoresis was used to evaluate whether a peptide–nanoparticle interaction takes place. Au/MUA nanoparticles show a band of relatively high electrophoretic mobility at pH 9, and the addition of only 5 µM VW05 leads to a complete loss in mobility of Au/MUA nanoparticles (Figure 6). On the other hand, the mobility of the Au/MUA nanoparticle band only slightly decreases when R1A3 is present; even a very high concentration of peptide R1A3 (200 µm) does not change this finding. All other peptides cause a stepwise decrease in Au/MUA nanoparticle mobility in a concentration-dependent manner. A peptide concentration of 100 µM is necessary to completely abolish electrophoretic mobility. To monitor the position of the peptide band next to Au/MUA nanoparticles and the presence of unbound peptide, the agarose gel was visualised by UV light at a wavelength of 254 nm. While an electrophoretic mobility of VW05 could be detected only for a high peptide concentration of 100 µM, the peptide band of R1A3 did not show any mobility. This effect could be explained by the formation of α-helical fibrils (vide infra) that are not able to enter the pores of the gel. The absence of a peptide band at 5 µM R1A3 is presumably due to the limited sensitivity of this technique. In contrast, R2A2 and all analogues with extended peptide length (see Supporting Information File 1) show a well-defined peptide band at a concentration of 10 µM with intensities increasing with peptide concentration.

Isothermal titration calorimetry (ITC) was used to determine the thermodynamic parameters as well as the binding constant for the assembly of Au/MUA nanoparticles (Table 1). By fitting with a one set of binding site mode the binding stoichiometry *N*, the binding constant *K*_B_, and the enthalpy Δ*H* can be directly obtained from the measured data whereas the entropy Δ*S* is calculated.

**Table 1:** Thermodynamic parameters for the aggregation of Au/MUA nanoparticles with peptides VW05, R2A2, R2A3, and R2A4 obtained by ITC.

	*N*	*K*_B_ [10^5^ M^−1^]	Δ*H* [kcal mol^−1^]	Δ*S* [cal deg mol^−1^]

VW05	20.2 ± 1.6	5.3 ± 1.7	3.5 ± 0.4	38
R2A2	17.9 ± 0.4	18.2 ± 5.9	−7.3 ± 0.2	5.8
R2A3	18.8 ± 1.4	6.3 ± 1.8	−4.9 ± 0.5	9.7
R2A4	17.3 ± 1.8	2.5 ± 0.8	−3.9 ± 0.5	11.5

Due to very weak or absent interactions of R1A3 or R2A5 with Au/MUA nanoparticles it was not possible to determine thermodynamic parameters. Surprisingly, the obtained binding constants are not in accordance with the observations made based on the UV–vis measurements. The shift in the absorption maximum obtained from UV–vis measurements for R2A2 is much smaller than that of VW05, but its binding constant of 18.2 **^.^** 10^5^ M^−1^ is about 3.5-fold higher than that of VW05, although the amount of presented charges is reduced in R2A2. The stepwise increase in peptide length induced a dramatic decrease of the *K*_B_ value. However, the *K*_B_ of R2A3 is slightly higher compared to VW05. Only a further increase in peptide length lead to a significant decrease in the binding constant compared to VW05. Although gel electrophoresis revealed a remarkable difference in the binding stoichiometry of VW05 versus R2A2 and its longer analogues, the binding stoichiometry is similar in all cases. The greatest *N* value is observed for the parent peptide VW05. Furthermore, major differences were observed with regard to the molar binding energy Δ*H*. First of all a positive molar binding energy for VW05 was determined while this was found to be negative for R2A2, R2A3, and R2A4. This can be explained by the different quaternary structure of peptides R2A2, R2A3, and R2A4 compared to VW05. The latter forms coiled-coil trimers that are refolded into α-helical fibers in the presence of Au/MUA nanoparticles, whereas R2A2 and its analogues are already present as fibers before interacting with nanoparticles, which results in a negative molar binding energy. The molar binding energy of these peptides can be directly correlated with their binding constants, as, for example, R2A2 shows the highest binding affinity, produces the greatest release of energy and has the smallest binding energy. With increasing peptide length, the binding affinity decreases, as does the release of energy, which is indicated by the increase in molar binding energy. Nevertheless, all peptide–nanoparticle interactions are entropically favored processes as entropy increases with along with binding energy.

### Morphological studies of peptide-induced nanoparticle assemblies

**Figure 7:**
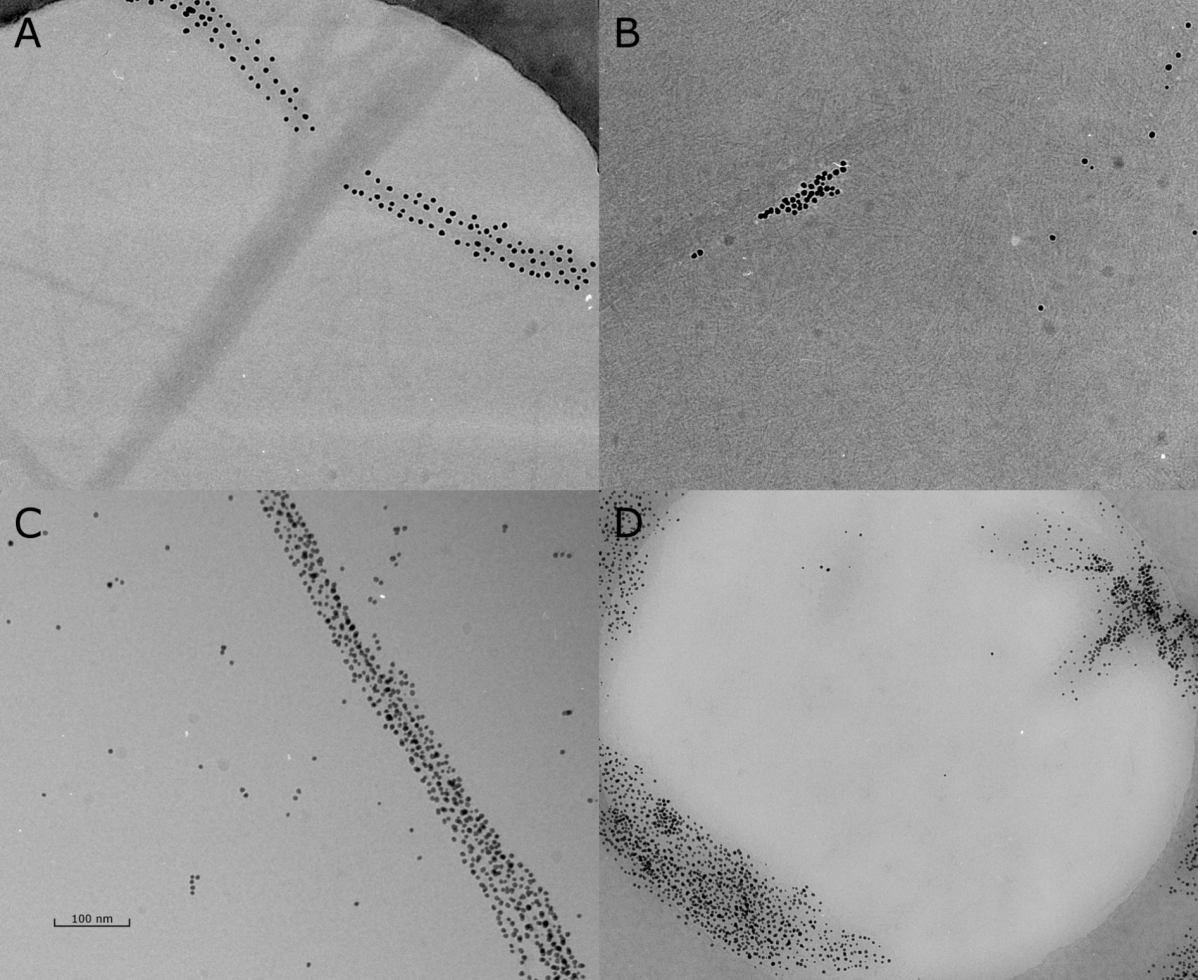
Cryo TEM images of 100 µM R2A2 and 0.05 µM Au/MUA nanoparticles at pH 9 at a defocus of (A) −1.2 µm, (B) −1.8 µm and (D) after pH switch from 11 to 9 at a defocus of −1.8 µm. (C) Cryo TEM images of 100 µM R2A2 and 0.4 µM Au/MUA nanoparticles at pH 9 at a defocus of −1.8 µM.

Cryo TEM was used to gain insight into the morphology of the peptide–nanoparticle aggregates. The concentration of peptide in all samples was 100 µM. It was already known that VW05 induces the aggregation of Au/MUA nanoparticles in a very disordered fashion. In the case of the modified analogues of VW05, the Au/MUA nanoparticles assemble in a completely different way. As can be seen in Figure 7A the nanoparticles are almost exclusively organized at the surface of the peptide fiber bundles. A similar effect was reported by Cherny and coworkers [41]. Since the nanoparticle concentration was 0.05 µM, an excess of peptide was present in solution. Thus it was expected that Au/MUA nanoparticles would be equally distributed on the surface of the peptide fibers. However, the nanoparticles were found to accumulate on the surface of larger fiber bundles, whereas some fiber bundles and especially single-peptide fibers remain unbound (Figure 7b). Upon increasing the nanoparticle concentration to 0.4 µM, unbound peptide could not be detected anymore whereas single and unbound nanoparticles are observed (Figure 7c). The obtained assemblies show a highly ordered adsorption of nanoparticles on the surface of the fiber in a three dimensional manner, which was supported by stereo Cryo TEM. Nevertheless, the decoration of peptides fibers with gold nanoparticles could only be observed for peptides that form fiber bundles. Peptides R2A4 and R2A5, which did not show bundle formation, led to a unordered nanoparticle aggregation comparable to those of VW05. Obviously, the single peptide fibers are more flexible and can surround the nanoparticles.

To evaluate whether the observed morphology is a result of the presence of peptide fibers even before the addition of nanoparticle, the pH of the peptide nanoparticle mixture was increased to pH 11 (Figure 7d). As previously reported for VW05 the change in pH results in disruption of the fibers and coiled-coil peptides are formed again. A subsequent decrease of the pH to 9 led to a dramatic change in the morphology of the peptide–nanoparticle assemblies. Whereas long and relatively thin nanoparticle-decorated fibers were observed prior to the change in pH, shorter and significantly brighter assemblies were obtained afterwards. However, even these assemblies do not exhibit the high level of organisation of nanoparticles on the fiber surface that had been previously observed. Moreover, a fully disordered nanoparticle accumulation was detected. These aggregates may be formed due to coiled-coil structures of the peptides since they appear to be very similar to those observed for VW05.

## Discussion

We have studied the effect of peptide length and net charge of coiled-coil-based sequences on their interaction with Au/MUA nanoparticles. Five analogues of the previously reported peptide VW05 were generated (Table 2) to study peptide-induced nanoparticle aggregation caused by electrostatic interactions. Due to the different number of presented arginine residues and length of the peptides the aggregation tendency and morphology of nanoparticles was modified. Peptide R1A3 presents one arginine residue in an f-position per 26-mer and was not efficient in interacting with nanoparticles. Peptides R2A2, R2A3, R2A4 and R2A5 efficiently interact with nanoparticles, although increasing the peptide length leads to a decrease in peptide–nanoparticle interactions as observed in lower binding affinities and reduced red-shifts of the absorption maximum. Obviously, a ratio of peptide length to presented charges not higher than 23.5 is required for specific interactions between coiled-coil peptides and nanoparticles.

**Table 2:** List of amino acid to arginine ratios for the sequences investigated here.

	amino acid residues	arginine residues	amino acids/arginine ratio

VW05	26	4	6.5
R1A3	26	1	26
R2A2	26	2	13
R2A3	33	2	16.5
R2A4	40	2	20
R2A5	47	2	23.5

Furthermore, the quaternary structure of the initial peptide plays an important role in nanoparticle assembly and the final morphology of nanoparticle–peptide aggregates. Whereas coiled-coil-forming peptides cause a more disordered nanoparticle assembly, fiber-forming peptides induce a well defined accumulation of the nanoparticles on their surface. Surprisingly, nanoparticles aggregate on the surface of larger fiber bundles but do not do so on single fibers. This may be the case because the amount of arginine residues in a single peptide fiber is insufficient to interact with nanoparticles. In addition, peptide fiber bundles were observed that do not have any nanoparticles attached to their surface. Apparently there is an unequal assembly of nanoparticles on the surface of the peptides.

The presence of preassembled peptide fibers has important consequences for the binding stoichiometry, the binding constant, and the molar binding energy of peptide–nanoparticle aggregation. Gel electrophoresis reveals a significant population of unbound peptide for R2A2, R2A3, and R2A4 at a peptide concentration of 10 µM, whereas a concentration of 100 µM of VW05 is required to observe unbound peptide. Nevertheless, peptide VW05 and its analogues have almost the same binding stoichiometry determined by ITC. This can be explained by considering the following points. Due to the fibril formation of R2A2, R2A3, and R2A4 Au/MUA nanoparticles interact with distinct positions on the surface of the peptide fiber. Cryo TEM images reveal, that an excess of peptide remains unbound in solution. In contrast, a coiled-coil peptide directly covers the nanoparticle surface and causes the assembly of two or more nanoparticles. Moreover the nanoparticles are covered with multiple peptide layers which is an ongoing process. In addition, the high local peptide concentration leads to α-helical fibril formation. As gel electrophoresis was carried out after an incubation time of 30 min, an excess of peptide could either bind to the nanoparticle surface or form fibrils. Thus, a higher binding stoichiometry is obtained by gel electrophoresis.

The differences in the quaternary structures of the peptides also lead to an unexpected observation in the binding constant data. Due to the greater number of arginine residues in VW05 it was expected that VW05 would have the highest binding constant. In fact the binding constant is 3.5-fold smaller compared to R2A2 and even the binding constant of R2A3 is slightly higher. Therefore it can be assumed that the fiber formation increases the binding constant significantly (Table 1). Compared to coiled-coil oligomers the α-helical peptide fiber has a highly ordered structure and the charge density is well localized on the surface of the fiber. Thus, the peptide nanoparticle interactions are more efficient due to direct interactions of nanoparticles with the surface of the fibers without any changes in the peptide structure. On the other hand, the peptide VW05 covers the surface of the nanoparticle, which results in an aggregation of the nanoparticles as well as a α-helical fiber formation of VW05. The latter one is a result of the high local peptide concentration on the surface of nanoparticles. However, peptide refolding reduces the binding constant *K*_B_. Thus, a well-defined peptide structure is crucial for increasing the binding constant. On the other hand, a larger number of arginine residues is not a prerequisite for increasing the binding constant.

Furthermore, the molar binding energies of the fiber forming peptides R2A2, R2A3, and R2A4 are different compared to the coiled-coil peptide VW05. In principle it can be assumed that the electrostatic interaction of peptide and nanoparticle is a reaction that releases heat which is indicated by a negative binding energy. Thus, the interaction between nanoparticle and fiber-forming peptide results in a negative binding energy which decreases along with the binding constant. During the VW05-induced aggregation of nanoparticles two reactions take place: 1) peptide-induced nanoparticle assembly, and 2) refolding of the coiled-coil peptide into α-helical fibers. But whereas the former is an exothermic reaction, the latter is an endothermic reaction: due to the fiber formation the coiled-coil structure has to be dissolved into single random-coil peptides. This is an energetically disfavoured process. Apparently the energy that is needed for dissolving the coiled-coil is higher than the energy that is delivered due to nanoparticle assembly and refolding into α-helical fibers. Thus, the whole reaction is endothermic.

Moreover, a pH switch has a dramatic effect on the observed nanoparticle assembly for the peptide–nanoparticle aggregates of R2A2, R2A3, and R2A4. Increasing the pH value to 11 causes a refolding of the peptide fibers into a coiled-coil structure. A subsequent decrease to pH 9 results in two concomitant reactions: the formation of peptide fibers and the nanoparticle aggregation. Cryo TEM reveals that both reactions occur approximately with the same reaction rates: on the one hand short and bright nanoparticle assemblies are detected that are presumably formed due to peptide fibers. On the other hand nanoparticle assemblies are observed that are very similar to those obtained for VW05 [32]. Apparently the nanoparticle decoration on the surface of the peptides is an intrinsic property of the fiber.

## Conclusion

The results presented herein demonstrate that the aggregation of Au/MUA nanoparticles depends not only on the number of presented arginine residues but also on the sequence length. The peptides studied here require a ratio not higher than 23.5 to specifically interact with oppositely charged nanoparticles. Thus the size of the peptide and finally the charge density plays an important role for its aggregation efficiency.

Furthermore, we could show that the quaternary structure of the peptide has important consequences for the formed nanoparticle assemblies, as well as for the thermodynamics of aggregation. First of all a peptide fiber leads to a well-defined nanoparticle aggregation on the surface, whereas soluble coiled-coil or random-coil peptides induce either an unstructured aggregation, or, in the case of random-coil peptides, no aggregation at all. Secondly, the peptide fiber with its well-defined presentation of charges causes an increase in the binding constant as well as in the binding energy. Thus, even a peptide with a lower number of charges can induce more rapid aggregation of nanoparticles if the peptide forms fibers. In contrast, nanoparticle aggregation induced by coiled-coil peptides even with a higher number of charges occurs more slowly.

## Supporting Information

File 1 Experimental procedures.

File 2 TEM images.
