# Supplementary material for: Impact of multivalent charge presentation on peptide–nanoparticle aggregation
Source: Beilstein J Org Chem. 2015 May 15;11:792–803. doi: 10.3762/bjoc.11.89 (PMC4463974; doi:10.3762/bjoc.11.89)
Supplement: File 1 — Experimental procedures. [file Beilstein_J_Org_Chem-11-792-s001.pdf]

Supporting Information File 1

for

Impact of multivalent charge presentation on peptide–nanoparticle aggregation

Daniel Schöne[1], Boris Schade[2], Christoph Böttcher[2] and Beate Koksch*[1]

Address: [1]Institute of Chemistry and Biochemistry - Organic Chemistry, Freie Universität Berlin, Takustr. 3, 14195 Berlin, Germany and [2]Electron Microscopy, Freie Universität Berlin, Fabeckstr. 36a, 14195 Berlin, Germany

Email: Beate Koksch* - beate.koksch@fu-berlin.de

*Corresponding author

Experimental procedures

Au/MUA nanoparticle synthesis [1,2]

Dodecanethiol (22 µL; 3.75 mmol) and $AuClPPh_3$ (20 mg; 0.04 mmol) were dissolved in benzene (3.2 mL). Borane-*tert*-butylamine complex (35 mg; 0.40 mmol) was added and the solution was stirred at room temperature for 2 hours. Methanol (10 mL) was added and the black precipitant was separated by centrifugation. The precipitate was washed 3 times with methanol (15 mL) and then dried under reduced pressure. The obtained powder was dissolved in 1.5 mL chloroform and slowly added to a solution of mercaptoundecanoic acid (MUA) (200 mg; 0.92 mmol) in THF (5 mL). After stirring for two days at room temperature a black precipitate was formed which was separated by centrifugation. The precipitate was washed with THF to remove the excess of MUA

and dried under reduced pressure. The obtained black powder was dissolved in Tris/HCl pH 11 (5 mL) and further purified by dialysis for three times.

Peptide synthesis and sample preparation

The peptides were synthesized by hand using standard Fmoc solid-phase peptide synthesis. Every amino acid was double coupled using 8 equiv HOBt, 8 equiv DIC and 8 equiv of the corresponding amino acid. To determine the peptide concentration the N-terminus was labeled with UV-active aminobenzoic acid (Abz). The peptides were cleaved from the resin using 95% TFA, 2.5% TIPS and 2.5% water, precipitated with ice cold ether and purified by reversed phase HPLC. The identification of the peptide was carried out by ESI-TOF.

Peptides were dissolved in freshly filtered Tris/HCl buffer (10 mM, pH 9). The peptide concentration was determined using UV–vis spectroscopy by measuring the absorbance at a wavelength of 314 nm. A calibration curve of glycine coupled to aminobenzoic acid (Abz-Gly) dissolved in the same buffer was used as a reference.

UV–vis spectroscopy

UV–vis spectra were measured on a CARY 50 Bio spectrophotometer (VARIAN, Darmstadt, Germany). The concentration of Au/MUA nanoparticles was calculated by measuring the absorption at 506 nm [3], was set to 50 nM. The final peptide concentration was set to 5 to 30 µM. UV–vis spectra were recorded right after addition of peptide in Tris/HCl buffer at pH 9 at room temperature using disposable semi-micro PMMA cuvettes (PlastiBRAND®, Brand GmbH, Wertheim, Germany). The spectra were taken from 450 to 750 nm in steps of 0.25 nm. All spectra were corrected by the buffer spectra.

Transmission electron microscopy (TEM)

5 µL of the peptide solution (concentration 100 µM) were placed on hydrophilized (1 minute plasma treatment at 8 W in a BALTEC MED 020) 400-mesh copper grids (BAL-TEC, Lichtenstein) that are carbon coated and supported with a collodium film. After 1 minute the supernatant fluid was blotted with a filter paper and 5 µL 2% PTA (pH 7.4) was added for 1 minute. After blotting with a filter paper the grid was left to air-dry. TEM images were taken with a Philips CM12 transmission electron microscope (FEI company, Oregon, USA) on Kodak SO-163 negative film. The accelerating voltage was 100 kV. A primary magnification of 60 k and a defocus of −500 nm was used.

Cryo preparation and cryo microscopy (Cryo TEM)

5 µL of the sample solution (100 µM peptide or peptide-nanoparticle mixture of various concentrations) were placed on hydrophilized (1 minute plasma treatment at 8 W in a BALTEC MED 020) perforated 200-mesh carbon coated copper grids (R1/4 batch of Quantifoil Micro Tools GmbH, Jena, Germany). After 1 minute the supernatant fluid was blotted with a filter paper until an ultrathin layer spanning the holes of the grid. The sample was immediately vitrified by soaking the grid into liquid ethane at its freezing point. The vitrified sample was stored under liquid nitrogen.

The grid was transferred into a Philips CM12 transmission electron microscope (FEI company, Oregon, USA) using a Gatan cryoholder (Gatan Inc., California, USA) and -stage (Model 626). The images were on Kodak SO-163 negative film. The accelerating voltage was 100 kV. A primary magnification of 60 k and a defocus of -1200 nm or -1800 was used.

CD spectroscopy

CD spectra were recorded on a J-810 spectrophotometer (Jasco GmbH, Gross-Umstadt, Germany) using a 2 mm quartz cuvette. All spectra are taken as the average of 3 single scan from 190 to 240 nm in steps of 0.5 nm at 20 °C. All spectra were corrected by the buffer spectra.

Isothermal titration calorimetry (ITC)

ITC measurements were carried out on a VP-ITC MicroCal instrument (MicroCal, LLC, Northampton, MA). The sample cell was loaded with the nanoparticle solution (200 µL of different concentrations in 10 mM Tris/HCl pH9). 38.5 µL of the peptide solution (different concentrations in 10 mM Tris/HCl pH 9) was titrated 20 times over a period of 5 seconds with an equilibration time of 150 seconds between every titration. The temperature was held constant at 20 °C.

Agarose gel electrophoresis

Agarose gel electrophoresis was carried out Gel XL Ultra V-2 Electrophoresis System (Labnet International, Windsor, Great Britain) using 0.5 % agarose gel in Tris/HCl buffer (10 mM, pH 9) with a accelerating voltage of 100 V. The electrophoresis was stopped after 15 to 20 minutes.

Dynamic light scattering (DLS)

DLS measurements were carried out on a Zetasizer Nano ZS analyzer™ with an integrated 4 mW He-Ne laser, λ= 633 nm (Malvern Instruments™ Ltd, U.K.). The peptides were dissolved in Tris/HCl buffer (10 mM, pH 9 or pH 11). Semi-micro PMMA cuvettes (PlastiBRAND®, Brand GmbH, Wertheim, Germany) at room temperature were used.

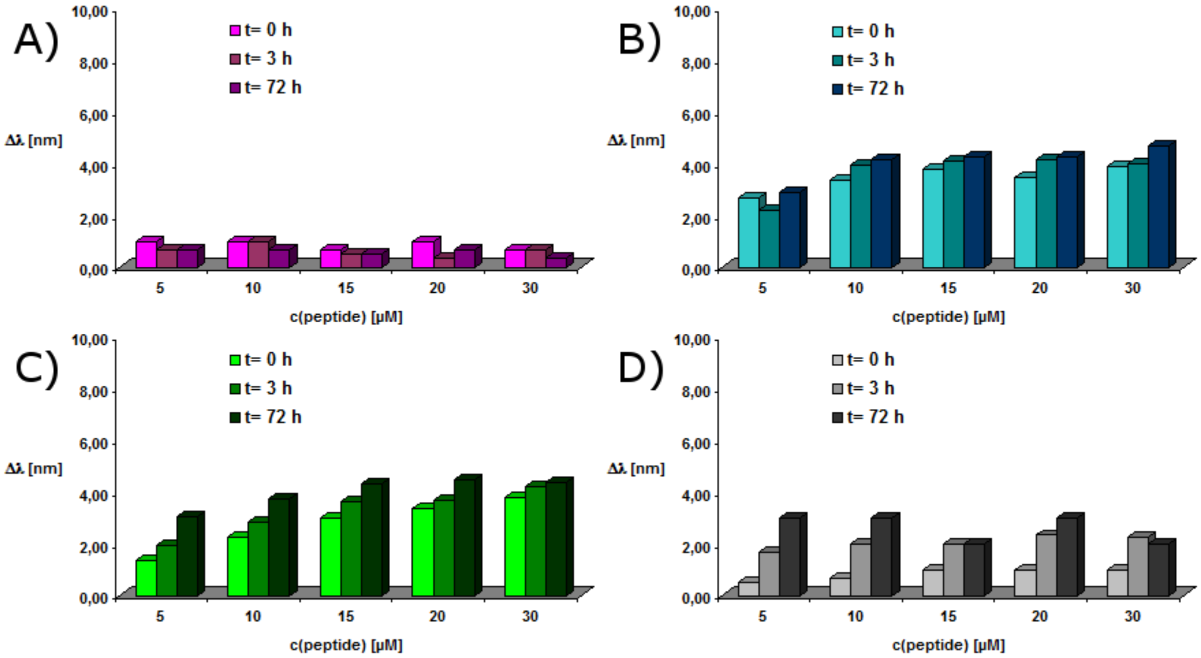

Figure S1: Time-dependent and concentration-dependent shift of the absorption maximum of UV–vis spectra of Au/MUA in the presence of R1A3 (A), R2A3 (B), R2A4 (C) and R2A5 (D).

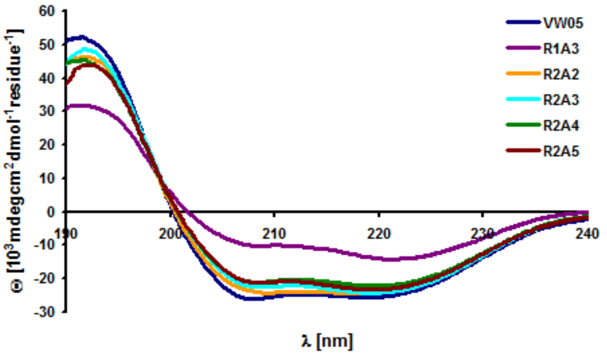

Figure S2: CD spectra of 30 μM peptide VW05, R1A3, R2A2, R2A3, R2A4 and R2A5 at pH 9 after 72 hours.

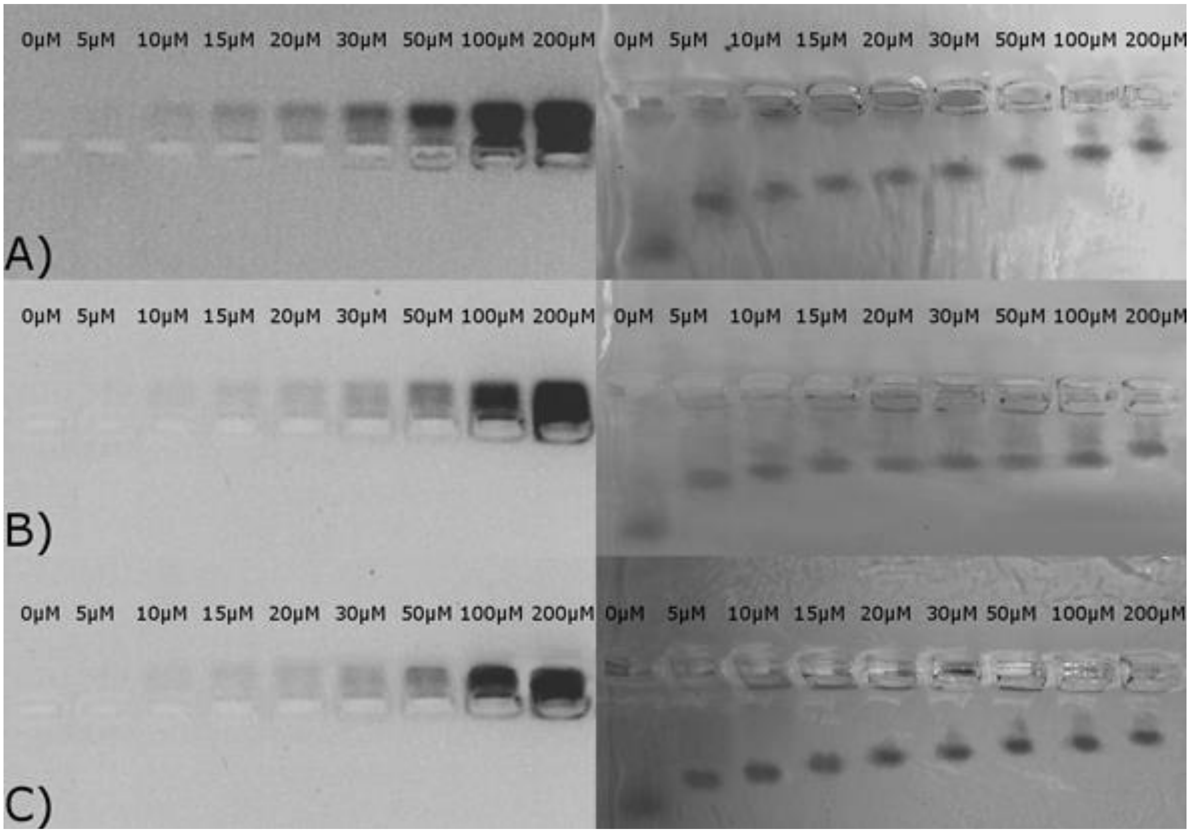

Figure S3: Agarose gel of R2A3 (A), R2A4 (B) and R2A5 (C) in the presence of 0.05 µM Au/MUA nanoparticles at pH 9 visualised by UV light (left) and visible light (right).

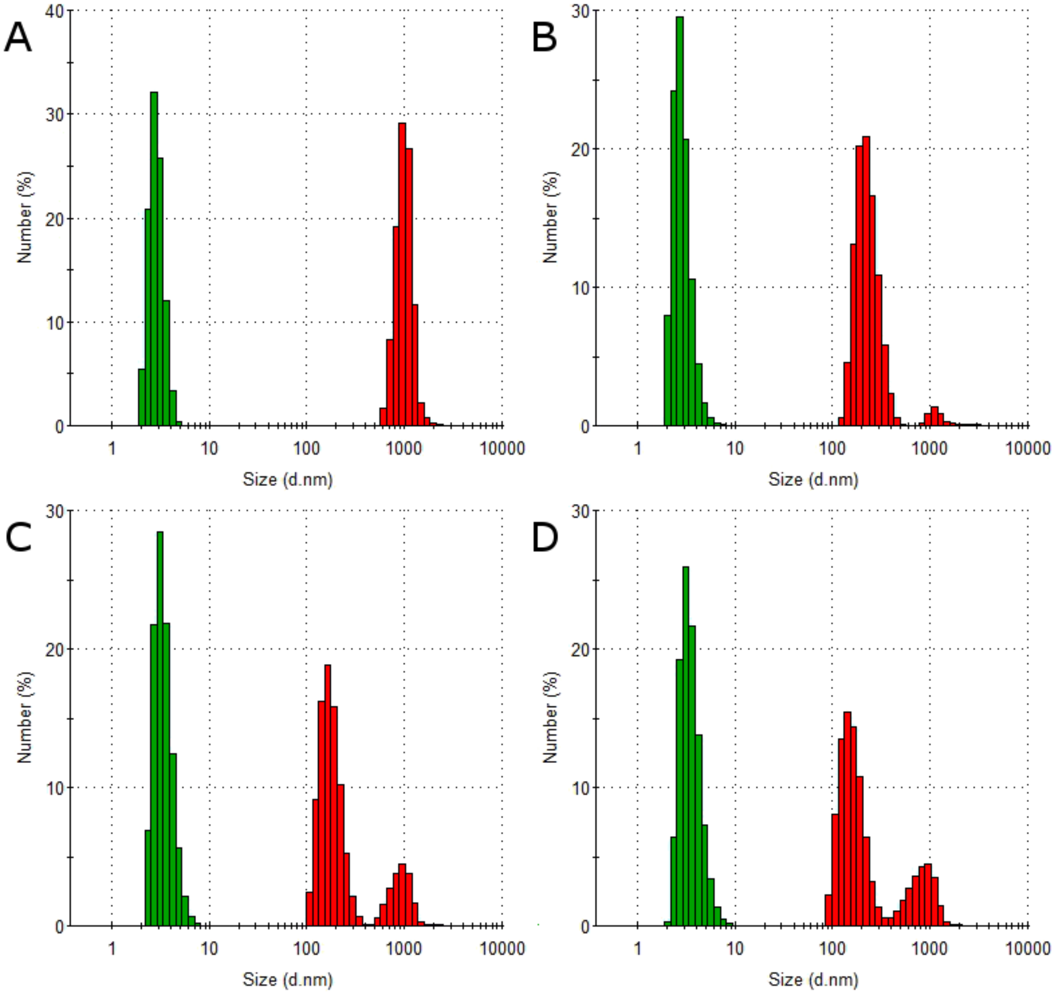

Figure S4: Dynamic light scattering of R1A3 (A), R2A3 (B), R2A4 (C) and R2A5 (D) at 15 μM at pH 9 (red) and pH 11 (green).

References:

1 Zheng, N.; Fan, J.; Stucky, G. D. *J. Am. Chem. Soc*. **2006**, *128*, 6550-6551.
2 Simard, J.; Briggs, C.; Boal, A. K.; Rotello, V. M. *Chem. Commun*. **2000**, 1943-1944.
3 Liu, X.; Atwater, M.; Wang, J.; Huo Q. *Coll. Surf. B* **2007**, *58*, 3-7.
