# Supplementary material for: Impact of multivalent charge presentation on peptide–nanoparticle aggregation
Source: Beilstein J Org Chem. 2015 May 15;11:792–803. doi: 10.3762/bjoc.11.89 (PMC4463974; doi:10.3762/bjoc.11.89)
Supplement: File 2 — TEM images. [file Beilstein_J_Org_Chem-11-792-s002.pdf]

Supporting Information File 2

for

Impact of multivalent charge presentation on peptide–nanoparticle aggregation

Daniel Schöne[1], Boris Schade[2], Christoph Böttcher[2] and Beate Koksch*[1]

Address: [1]Institute of Chemistry and Biochemistry - Organic Chemistry, Freie Universität Berlin, Takustr. 3, 14195 Berlin, Germany and [2]Electron Microscopy, Freie Universität Berlin, Fabeckstr. 36a, 14195 Berlin, Germany

Email: Beate Koksch* - beate.koksch@fu-berlin.de

*Corresponding author

TEM images

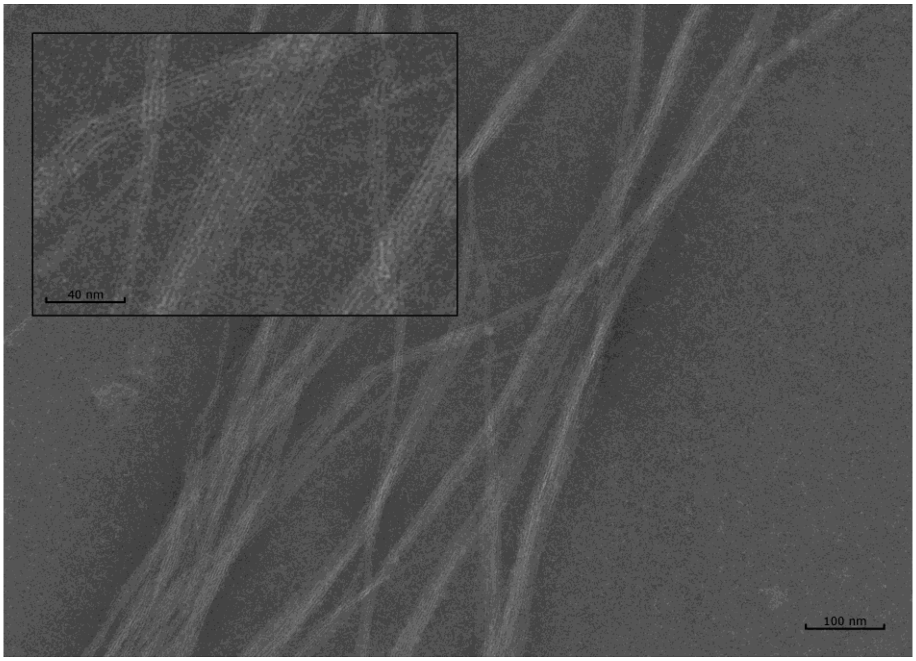

Figure S5: TEM of 100 µM R1A3 in 10 mM Tris/HCl buffer pH 9. Sample was negative stained with 2 % PTA, defocus - 0.5 µm.

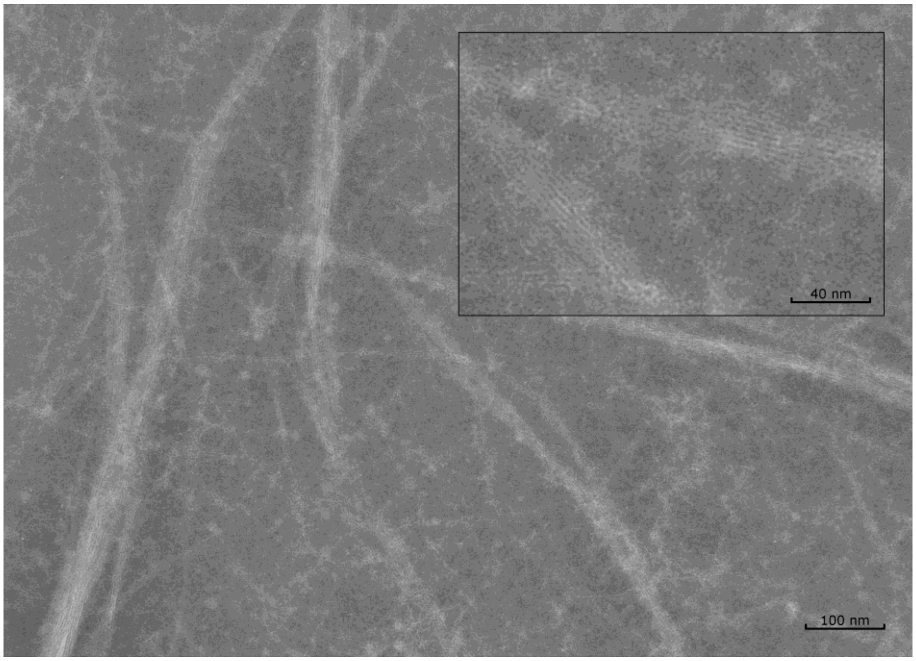

Figure S6: TEM of 100 µM R2A3 in 10 mM Tris/HCl buffer pH 9. Sample was negative stained with 2 % PTA, defocus - 0.5 µm.

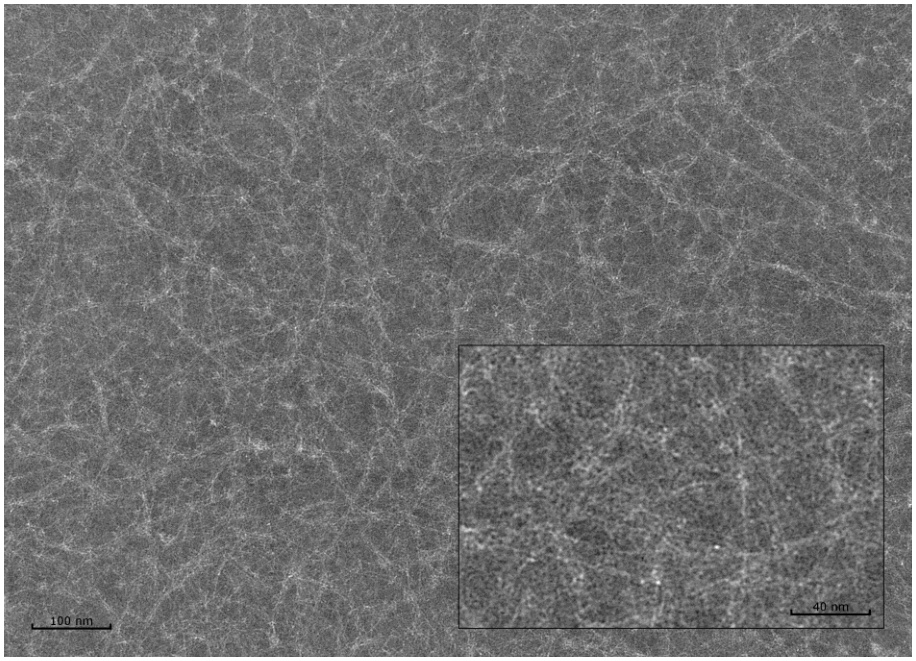

Figure S7: TEM of 100 µM R2A4 in 10 mM Tris/HCl buffer pH 9. Sample was negative stained with 2 % PTA, defocus - 0.5 µm.

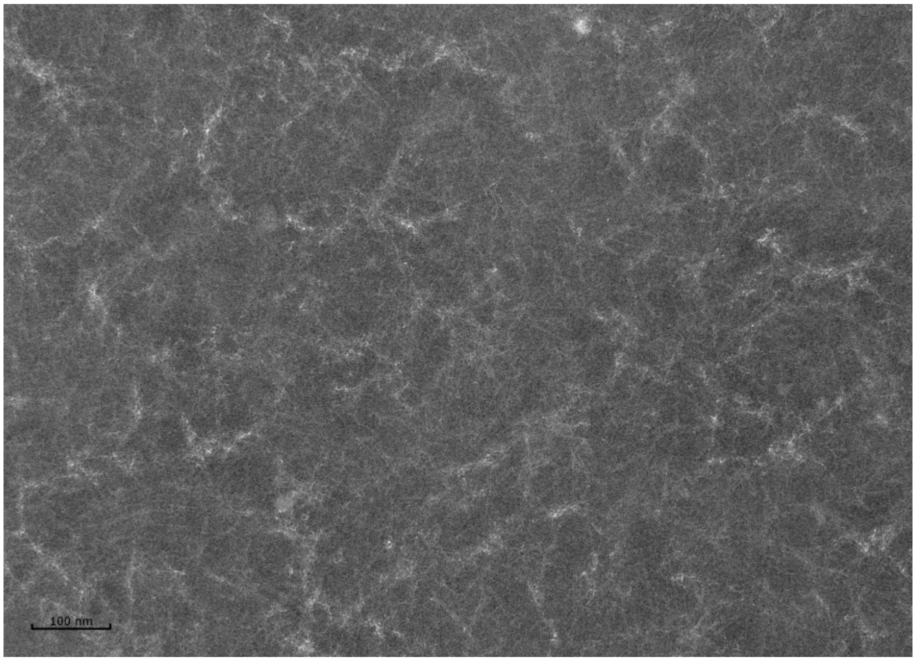

Figure S8: TEM of 100 µM R2A5 in 10 mM Tris/HCl buffer pH 9. Sample was negative stained with 2 % PTA, defocus - 0.5 µm.

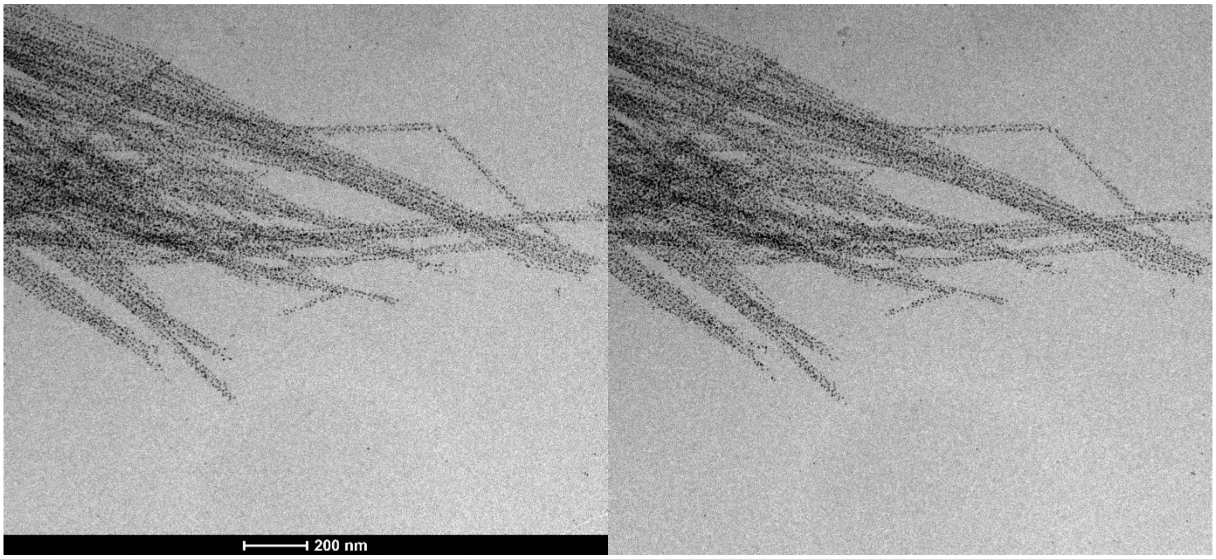

Figure S9: Stereo Cryo TEM of 100 µM R2A2 and 0.4 µM Au/MUA nanoparticles in 10 mM Tris/HCl buffer pH 9.
